# Noble Metal-Free Hierarchical ZrY Zeolite Efficient for Hydrogenation of Biomass-Derived Levulinic Acid

**DOI:** 10.3389/fchem.2021.725175

**Published:** 2021-10-12

**Authors:** Di Hu, Hong Xu, Zuotong Wu, Man Zhang, Zhiyue Zhao, Yuchen Wang, Kai Yan

**Affiliations:** Guangdong Provincial Key Laboratory of Environmental Pollution Control and Remediation Technology, School of Environmental Science and Engineering, Sun Yat-sen University, Guangzhou, China

**Keywords:** noble metal-free, ZrY, hydrogenation, levulinic acid, gamma-valerolactone

## Abstract

Developing a low-cost and robust catalyst for efficient transformation of biomass-derived platform chemicals plays a crucial role in the synthesis of future transportation fuels. Herein, a post-synthetic strategy was employed to develop a noble metal-free and robust ZrY zeolite catalyst, which is efficient for the hydrogenation of biomass-derived levulinic acid (LA) into biofuel γ-valerolactone (GVL), whereas over 95% yield of GVL was achieved in 10 h at 220°C. The effects of acidic properties from ZrY catalysts and various reaction parameters on the catalytic performance were then discussed in detail. Subsequently, different characterization tools were used to compare the difference and relationship of structure activity between the fresh and spent ZrY catalysts. It was found that acidity and the metal–support interaction were important for the direct synthesis of GVL. This work provides a guideline to design a noble metal-free catalyst for high-value utilization of biomass-derived sources.

## Introduction

With the rapid consumption of fossil resources and the raising concern on the environmental issues, the development of efficient technologies for the conversion of renewable biomass into platform molecular and valuable chemicals has gained much attention around the world (Kunkes et al., 2008; Alonso et al., 2013; Wang et al., 2014). So far, various value-added chemicals (e.g., 5-hydroxymethylfurfural, lactic acid, levulinic acid (LA), γ-valerolactone (GVL), and 2,5-dimethylfuran) have been produced from naive biomass (Liu and Zhang, 2015; Xue et al., 2018). Among these valuable chemicals, GVL has been considered as a versatile platform chemical that can be used as a fuel additive, a solvent for biomass processing, and a precursor for the production of alkanes and valuable chemicals (Feng et al., 2019; Li et al., 2019; Winoto et al., 2019; Yu et al., 2019).

Over the last few decades, several methodologies for the production of GVL from various resources have been developed and reported (Moustani et al., 2018; Wang et al., 2018; Winoto et al., 2019). Among the various strategies, the heterogeneous hydrogenation of LA and its esters appeared more promising using various metal catalysts, such as noble metal (Pd (Yan et al., 2017a), Ru (Luo et al., 2014; Kuwahara et al., 2017), Au (Zhu et al., 2016), Pt (Luo et al., 2016), and Re (Yu et al., 2019)) and non-noble metal (Ni (Singh et al., 2018) and Cu (Hengne and Rode, 2012) ) catalysts. The noble metal catalysts seem to be the more active and selective candidates for the production of GVL from LA hydrogenation, due to their high intrinsic abilities in the activation of the C=O bond (Luo et al., 2014; Kuwahara et al., 2017; Yan et al., 2017b). However, the high cost and complex synthesis production limit their practical application. Besides, the catalytic hydrogenation procedure often associates with the use of high H_2_ pressure, noble metals, and unsatisfied catalyst stability, restricting the actual utilizations. Developing noble metal-free candidates with high performance in the selective hydrogenation of LA is highly desired. Recently, Singh et al. (Singh et al., 2018) synthesized Ni/NiO catalyst for the catalytic hydrogenation of LA, and it was found that the acidity property plays an important role in the conversion of LA and ML to GVL with 94% yield. Zhang et al. (Zhang et al., 2018) reported that the geometric and electronic effects between Cu and Ag were responsible for the good activities of CuAg/Al_2_O_3_ catalyst in the synthesis of GVL with approximately 100% yield. Recently, we also found the metal effect (Yi et al., 2020; Xu et al., 2021) and solvent effect (Xu et al., 2019) in tuning the hydrogenation of LA.

Although these abovementioned advanced works have been reported, few works have concentrated on the direct use of noble metal-free oxides for the hydrogenation of LA. On the other hand, the catalytic transfer hydrogenation of LA using Zr-based catalysts such as ZrO_2_ has been reported to effectively produce GVL, whereas ZrO_2_ was proposed as the active center (Li et al., 2017). The strong metal–support interaction between Ru and nanotetragonal ZrO_2_ (Cao et al., 2017) made great contribution. The high porosity and acidity of the as-prepared catalyst facilitate the LA conversion to GVL. Zeolites were widely used in catalysis both as a catalyst and support due to the tunability of the structure and acidity. These findings inspired us to utilize the acidic property of ZrO_2_ and the strong metal–support interaction to overcome the considerable technical barrier.

Herein, we report the efficient hydrogenation of LA to GVL using the hierarchical noble metal-free ZrY catalysts (ZrO_2_ supported on NaY), whereas the ZrY catalyst was prepared using the post-synthetic method. Combination analysis tools were used to study the physical properties of these fabricated catalysts and understand the relationship between structure and activities. These fabricated catalysts were screened in the selective hydrogenation of LA, and various reaction parameters on the activities were investigated. Besides, catalyst stability was evaluated, and the proper reaction mechanism was proposed. In particular, the noble metal-free ZrY catalysts developed in this work are facile and alternative for the high-value upgrading of various biomass-derived chemicals.

## Materials and Methods

### Catalyst Preparation

NaY with an Si/Al ratio of 5.4 was purchased from Nankai Catalyst Plant. NaY_meso_ was prepared by treating NaY which contains uniform micropores. Typically, 3.5 g of NaY and ethylenediamine-tetraacetic acid aqueous solution (50 ml, 0.035, 0.07, and 0.105 mol L^−1^) were added to a flask. The mixture was refluxed and stirred at 373 K for 6 h. Then, the solid product was filtered and dried for subsequent NaOH treatment. For the alkali treatment process, 1.7 g of solid powder was added to the NaOH aqueous solution (50 ml, 0.4 mol⋅L^−1^) at 338 K for 0.5 h. The formation of NaY_meso_ was then filtered, dried, and calcined in air at 823 K for 6 h, and denoted as Y_0.5_, Y_1_, and Y_1.5_. In order to functionalize the NaY_meso_, nitrate solutions of Ce^3+^ (50 ml, 0.2 mol L^−1^) and zirconium acetate (50 ml, 0.1, 0.2, and 0.3 mol L^−1^) were, respectively, used to exchange 1.0 g of NaY_meso_ at 353 K for 12 h. The filtered products were dried and calcined in air at 823 K for 6 h, and then the powder denoted as Ce_1_Y_1_ and Zr_x_Y_y_ were obtained (x and y was equal to 0.5, 1, and 1.5). Also, the synthesis procedures of hierarchical HZSM-5 were referred to the previous literatures (Wang et al., 2017), and Zr_1_HZSM-5 was produced from zirconium acetate (50 ml, 0.2 mol L^−1^) treatment and calcination. ZrY was directly synthesized from NaY exchanging with zirconium acetate and calcination step. ZrO_2_ was obtained directly from the calcination of zirconium acetate at 823 K for 6 h. All the reagents were bought from Macklin and used without any pretreatment. The used Zr_1_Y_1_ catalyst was recollected after five consecutive runs.

### Catalytic Tests

The catalytic reaction was performed in an electrically heated high-pressure autoclave with a magnetic stirring system at 600 rpm, during 10 h at 220°C and 3 MPa H_2_, using 10 ml of 1,4-dioxane, 4 mmol of LA, and 100 mg catalyst, and then the gas was released carefully after being cooled to room temperature. The reaction mixture was centrifuged for separation of slurry and supernatant. The slurry was washed several times with water and ethanol for a subsequent cyclic test and further analyzation; the supernatant was filtered through a 0.22-µm syringe filter for analyzing by gas chromatography (Techcomp GC7900) equipped with a capillary column (TM-FFAP) and an FID detector.

The concentration of LA and GVL was calculated by standard calibration curves. The conversion of LA and selectivity of GVL were determined as follows:

$$\mathrm{LA}\;Conversion\left(\%\right)=1-\left(\frac{Remaining\;amount\;of\;LA}{Initial\;amount\;of\;LA}\right)\times 100\%,$$ (1)

$$GVL\;Selectivity\left(\%\right)=\left(\frac{Amount\;of\;GVL}{Sum\;amount\;of\;products}\right)\times 100\%.$$ (2)

### Catalyst Characterization

X-ray diffraction (XRD) of all samples for the crystal phase analysis was recorded on a Rigaku Ultima IV diffractometer. Scanning electron microscope (SEM) images were obtained with a HitachiE-3500 to elucidate the morphologies of the samples. Transmission electron microscopy (TEM) images were obtained with an accelerating voltage of 300 kV (TEM, TF20). Mapping was employed to explore the existence and the distribution of elements in the sample. Temperature-programmed desorption (TPD) experiments were performed using an AutoChem II 2920 V5.02 chemical adsorption instrument with a thermal conductivity detector (TCD). X-ray photoelectron spectra (XPS) were performed on an Escalab 250 X-ray photoelectron spectrometer (Thermo Fisher Scientific, USA). The thermal behavior of fresh and used catalysts were tested by TGA5500 from room temperature to 800°C under air atmosphere, with a heating rate of 10°C/min. Nitrogen sorption isotherms of samples were performed at −196°C on Quantachrome instrument under nitrogen atmosphere.

## Results and Discussion

### Characterization Results of the Hierarchical ZrY Catalysts

**FIGURE 1:**
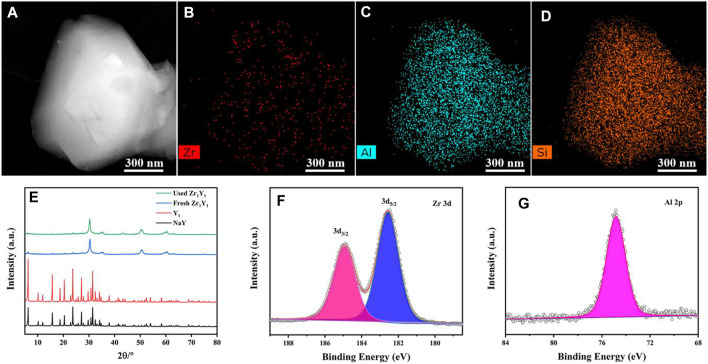
TEM images of Zr_1_Y_1_ catalyst **(A)**, corresponding EDS mapping patterns **(B–D)**, XRD patterns of different samples **(E)**, XPS spectra of the Zr_1_Y_1_ catalyst **(F)**, Zr 3d, and **(G)** Al 2p.

The characterization results for the TEM image and elemental distribution patterns of Zr, Al, and Si in the Zr_1_Y_1_ catalyst are presented in Figures 1A–D. The mapping results demonstrated the homogeneous dispersion of Zr (Figure 1B), Al, and Si uniformly throughout the particles, indicating that ZrO_2_ was homogeneously immobilized onto the surface of the NaY_meso_ support with no severe aggregation. The effect of various parameters on the synthesis process (dealumination and Zr incorporation) on the NaY zeolite crystal structure was investigated by XRD, as shown in Supplementary Figure S1 and Figure 1E. The treated NaY zeolite exhibited its inherent crystal structure, and the incorporation of Zr atoms into NaY_meso_ did not affect its crystal structure but displayed the similar XRD pattern as the parent NaY. Importantly, there is apparently appearance of the typical tetragonal ZrO_2_ (JCPDS: 50-1,089) peaks at 30.3°, 50.4°, and 60.2° (Huang et al., 2018), suggesting the successful mobilization of Zr atoms onto the framework. In addition, there is no other type of diffraction peak for the Zr_x_Y_y_ catalyst, indicating the existence of NaY and ZrO_2_ for all samples. And there is a dilute effect in the adjusting of the NaY and Zr salt dosage. For the Zr_1_Y_1.5_ catalyst, the crystallinity was partially damaged, and the interaction between support and ZrO_2_ species was lower than that in the Zr_1_Y_1_ sample. Also, the fresh and used Zr_1_Y_1_ catalysts exhibited the same XRD patterns, indicating the strong interaction between NaY support and loaded ZrO_2_ species. Supplementary Figure S3 illustrated the XPS survey image, and Figures 1F,G displayed the XPS spectra of Zr 3d and Al 2p in the Zr_1_Y_1_ catalysts. Regarding the Zr 3d spectra, the Zr_1_Y_1_ catalyst exhibited two distinct peaks at 182.5 and 184.9 eV corresponding to Zr 3d_5/2_ and Zr 3d_3/2_, respectively (Li et al., 2017; Liu et al., 2017). These further confirmed the formation of the combining ZrO_2_ with stable zeolites in a single catalytic system.

The porosity information was analyzed by BET as shown in Supplementary Figure S4 and Supplementary Table S1. The dealuminized Y_1_ zeolite catalyst displays a typical type I adsorption isotherm superficial; from the Supplementary Figure S4 inset picture, a hysteresis loop can be seen, and an average diameter of 1.54 nm was obtained (Crisci et al., 2010), which is higher than that of NaY, indicating the dealuminization effect. During the dealuminization process, the main structure was maintained, and the newly formed vacancy could act as the doping sites for the following step. After loading Zr onto the support, a type IV isotherm with H4 type hysteresis loops was recognized (Ahn et al., 2018), the specific surface area is 192 m^2^/g, and the average pore size is 2.31 nm, which shows clearly that the mesoporous structure existed. And this will facilitate the transportation of LA and GVL during the reaction process.

Ammonia temperature programmed desorption was further employed to investigate the acidity of NaY support, the as-prepared catalyst, and used catalyst (Supplementary Figure S5) (Delidovich et al., 2014; Körner et al., 2018). The amount of different acidic sites was quantified and listed in Table 1. It can be found that NaY kept a desorption peak at around 153°C, indicating the existence of weak acid sites, the acid amount was 2.59 mmol/g, and there is no other type of acid sites detected. The desorption peak at around 276 and 593°C for the Zr_1_Y_1_ samples represented moderate and strong acid sites. Also, the used Zr_1_Y_1_ showed dominate acid sites are moderate and strong acid sites, with just 0.16 mmol/g weak acid sites. These results confirmed that the parent NaY only held weak acid sites. As our previous work reported (Xu et al., 2019; Yi et al., 2020), the acidity property plays an important role in the conversion of LA, and this work further confirmed that the moderate and strong acid sites can promote the hydrogenation of LA to GVL, whereas the weak acid sites have a weak ability to adsorb H_2_ and LA (Yan et al., 2015).

**TABLE 1:** Acidity properties of NaY, Zr_1_Y_1_, and used Zr_1_Y_1_ catalysts.

Catalysts	Weak acidity		Moderate acidity		Strong acidity	
	T_d_ (°C)	Amount (mmol/g)	T_d_ (°C)	Amount (mmol/g)	T_d_ (°C)	Amount (mmol/g)
NaY	153	2.59	-	-	-	-
Zr_1_Y_1_	123	0.68	276	0.68	593	0.33
Used Zr_1_Y_1_	100	0.16	337	0.92	547	0.06

**FIGURE 2:**
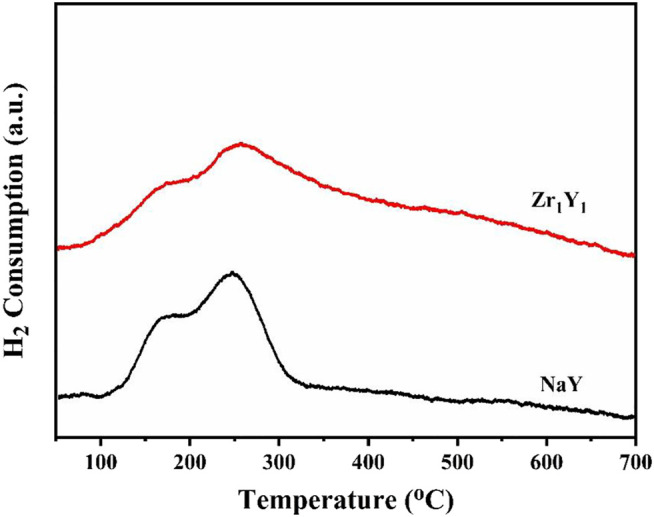
H_2_-TPD profiles of the NaY and Zr_1_Y_1_ catalysts.

Figure 2 shows the H_2_ temperature programmed desorption (H_2_-TPD) profiles of the pristine and modified catalysts. For Zr_1_Y_1_ catalyst, the H_2_-TPD profile displays two H_2_ desorption peaks centered at 174 and 257°C, respectively, in the range of 50–700°C. The high-temperature peak was attributed to the H_2_ adsorbed in the subsurface and the spillover. The Zr_1_Y_1_ catalyst exhibits twice the H_2_ uptake ability than that of the pristine NaY, which is 167 and 80.8 μmol g_cat_^−1^, indicating that the Zr_1_Y_1_ is more efficient for the adsorption and dissociation sites of H_2_, which is further verified by the catalytic hydrogenation performance of the catalysts. The as-prepared Zr_1_Y_1_ catalyst displayed more easily accessible ZrO_2_ acidic sites and the higher affinity with the H_2_ resources, which is beneficial for the selective hydrogenation of LA in a bench reactor.

### Catalytic Hydrogenation of LA

**FIGURE 3:**
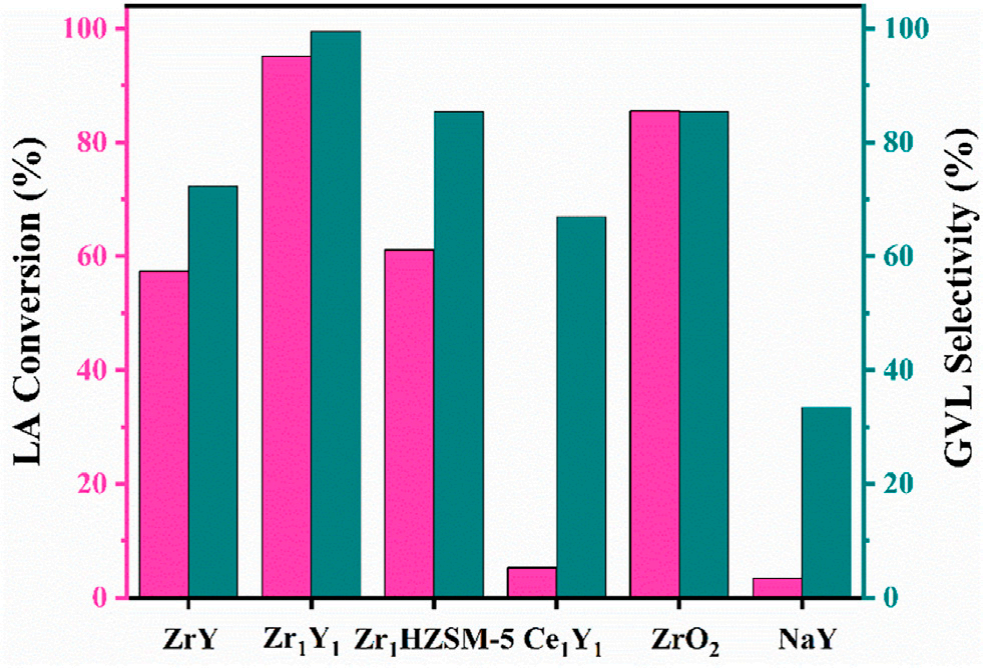
Catalytic performance of LA hydrogenation with various catalysts.

The as-prepared catalysts were emulated for the selective hydrogenation of LA under the same conditions. As revealed in Figure 3, Zr_1_Y_1_ catalyst exhibited the highest conversion of 95% and GVL selectivity of 99% among the presented catalyst. The hierarchical structure and suitable acidity offered outstanding transportation properties and active centers for the reaction. For example, it showed a negligible activity for the NaY support (less than 5%), due to the lack of active sites. The sole usage of ZrO_2_ catalyst was also evaluated, and the hydrogenation reaction was quite active and can achieve 85%; however, the activity in the second run decreased severely, which was probably owing to the strong adsorption ability in leading to the carbon deposit formation. When the acidic ZrO_2_ was directly loaded onto the microporous structure of Y zeolite by the exchanging process, 57% conversion of LA and 72% selectivity to GVL were obtained. Due to the lack of the modification step, a weak interaction between ZrO_2_ and NaY was formed. In contrast, the two different metals supported on hierarchical Y zeolite catalysts Zr_1_Y_1_ and Ce_1_Y_1_ displayed a thorough difference in the performance, and the CeO_2_ was unable to catalyze the conversion of LA. Also, another type of hierarchical zeolite HZSM-5 was chosen as a support for the synthesis of Zr_1_HZSM-5 catalyst; apparently, the mesopore-contained Zr_1_HZSM-5 catalyst could facilitate the hydrogenation reaction rate. These facts suggested that both the acidity of catalyst and the mesoporous structure can increase the catalytic activity in the direct hydrogenation of LA. Also, the strong interaction between metal and support is crucial for this process.

To optimize the catalytic performance of the modified Zr_x_Y_y_ catalyst in the selective hydrogenation of LA, the dealuminization rate and loading amount of ZrO_2_ varied, and their influence on the hydrogenation of LA is shown in Supplementary Figures S7,S8. When the dealuminization rate of NaY was changed from 50 to 150%, the yield of GVL displayed a volcano-type behavior with a highest yield of GVL at 100%, which is 73, 95, and 89%, respectively. This can be attributed to the fact that the dealuminization rate of NaY matrix need to be controlled in a proper range. An enhancement in the dealuminization rate beyond 100% could lead to the breakdown of the NaY structure; this can be proved by the decrease in the intensity of Zr_1_Y_1.5_ in Supplementary Figure S1c. In another case, the optimum loading of ZrO_2_ on the hierarchical NaY_meso_ was found to be Zr_1_Y_1_. When the ZrO_2_ loading amount was lower than 100%, the GVL yield decreased to 72%. While the amount of ZrO_2_ was higher than 100%, the GVL yield was 88%, which also indicated the lack of active sites and pore blockage. Hence, an appropriate amount of the modification agent and Zr salt were used for the Zr_1_Y_1_ catalyst, and the changes in the physicochemical offer great support for the LA hydrogenation process.

**FIGURE 4:**
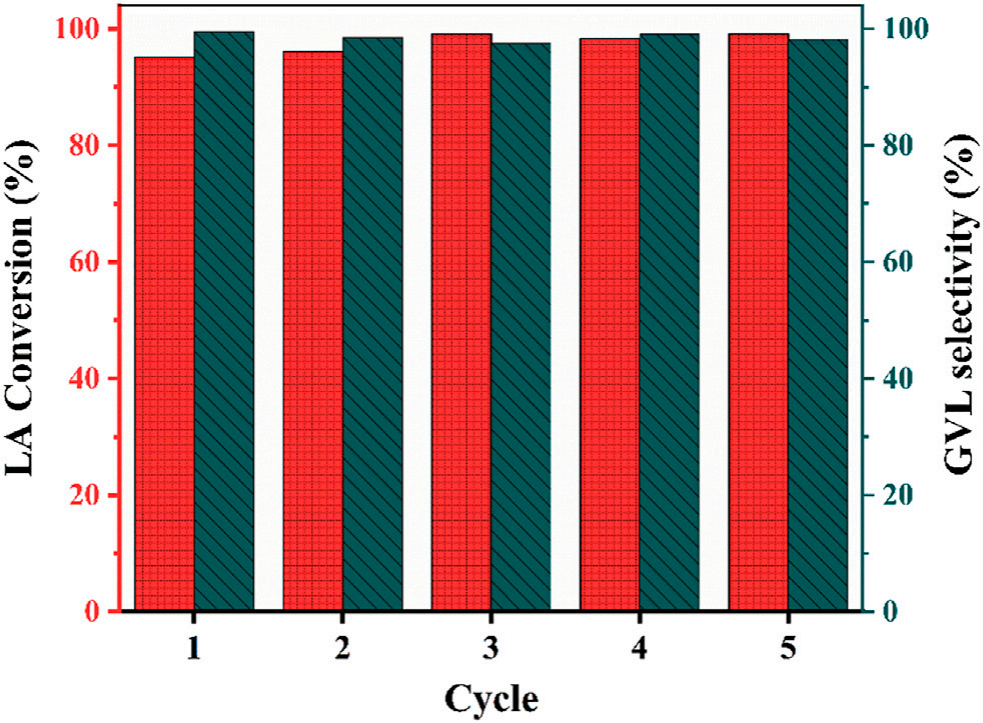
Cyclic stability test of the Zr_1_Y_1_ catalyst.

Finally, the recyclability experiments were tested to evaluate the catalytic performance under the same reaction conditions. The cyclic results for the hydrogenation of LA to GVL using Zr_1_Y_1_ catalyst are shown in Figure 4, demonstrating that the LA hydrogenation and selectivity of GVL remained steadily even after five cycles. There is no decrease in all the runs, while it has ∼4.9% coke deposition formed as revealed in Supplementary Figure S6 (Zhao et al., 2018). For the zeolite type of catalyst, the coke deposition can be removed by the calcination step (Yi et al., 2020), and the Zr_1_Y_1_ catalyst was directly used after calcination at 550°C without any reducing treatment. Figure 1E displayed the XRD patterns of fresh and used Zr_1_Y_1_ catalysts, there are no obvious changes, indicating there is no severe ZrO_2_ species leaching process. In general, the Zr_1_Y_1_ catalyst was proven to be stable. Thus, the specific rate of reaction is about 4 mmol_GVL_ g_cat_^−1^ h^−1^ in the consecutive runs. This was due to the ZrO_2_ uniform dispersed on the treated Y zeolite, effectively preventing agglomeration and enhancing the moderate and strong acidity of the weak acidity and micropore structure Y zeolite. And, with the reuse of the Zr_1_Y_1_ catalyst, the considerable amount of strong acidity was converted to moderate acidity due to the agglomeration process on the surface. In addition, the Zr_1_Y_1_ catalyst was directly separated from solutions with filtering and dried in the oven, and then it can be reused without any calcination, making it attractive for practical usage.

**SCHEME 1:**
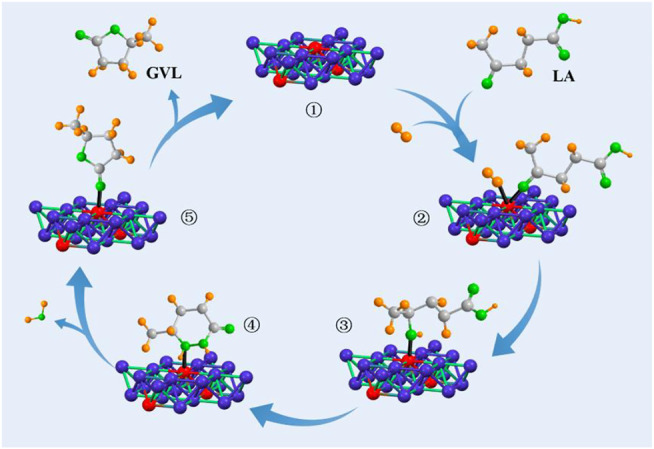
Proposed mechanism for the direct hydrogenation of LA to GVL over the Zr_1_Y_1_ catalyst.

Based on the catalytic performance, a plausible Meerwein–Ponndorf–Verley (MPV) reduction mechanism for the hydrogenation of LA to GVL was proposed through a catalytic cycle involving a six-member ring transition state. As shown in Scheme 1, molecular H_2_ and the ketone carbonyl group of LA were adsorbed to the ZrO_2_ sites. Then, 4-hydroxyvaleric acid was formed to generate state 2. Between intermediates 2 and 3, the two hydroxyl groups were linked together to create a six-membered ring-like structure, and ether was formed with the acidity catalyze, and 1 mole H_2_O was discharged during the reaction. After that, GVL and the bond between the O–O linker were released. Each step in the whole process was believed to be reversible, and the reaction was driven by the thermodynamic properties of the intermediates (Wang et al., 2018). The newly formed 4-hydroxypentanoic was not stable and readily converted to target product GVL.

## Conclusions

In summary, we have successfully developed noble metal-free Zr_1_Y_1_ catalysts by a post-synthetic strategy, which is efficient for the hydrogenation of LA into biofuel GVL. As a result, the conversion of LA and selectivity of GVL reached 95 and 99%, respectively, under the reaction conditions of 220°C and 3 MPa H_2_ pressure in 1, 4-dioxane solvent. The performance was comparable with previously reported candidates. Besides, it was found that acidic properties and the metal–support interaction play important roles in the synthesis of GVL. Moreover, these as-prepared ZrY catalysts maintained stable performance even over five consecutive runs, indicating the promising applications for the selective upgrading of other biomass-derived chemicals.

## Data Availability

The original contributions presented in the study are included in the article/Supplementary Material; further inquiries can be directed to the corresponding author.
