# Supplementary material for: Noble Metal-Free Hierarchical ZrY Zeolite Efficient for Hydrogenation of Biomass-Derived Levulinic Acid
Source: Front Chem. 2021 Oct 12;9:725175. doi: 10.3389/fchem.2021.725175 (PMC8546667; doi:10.3389/fchem.2021.725175)
Supplement: Supplementary file 1 [file DataSheet1.docx]

Supplementary Material

Noble-metal free hierarchical ZrY zeolite efficient for hydrogenation of biomass-derived levulinic acid

Di Hu^1^, Hong Xu^1^, Zuotong Wu^1^, Man Zhang^1^, Zhiyue Zhao^1^, Yuchen Wang^1^, Kai Yan^1*^

^1^Guangdong Provincial Key Laboratory of Environmental Pollution Control and Remediation Technology, School of Environmental Science and Engineering, Sun Yat-sen University, 135 Xingang Xi Road, Guangzhou, P. R. China

*** Correspondence:**Kai Yan
[yank9@mail.sysu.edu.cn](mailto:yank9@mail.sysu.edu.cn)

**Catalyst characterization**

X-ray diffraction patterns (XRD) were recorded on a diffractometer operating at 40 kV and 40 mA with monochromatic Cu-Kα radiation (λ= 0.15418 nm), the scan range was from 5^o^ to 90^o^. with a scan rate of 10^o^ min^-1^. High-angle annular dark-field scanning transmission electron microscopy (HAADF-STEM) images were recorded on a JOEL-JEM 2100 STEM equipped with an energy dispersive X-ray (EDX) spectroscope, operating at 200 kV. Temperature-programmed desorption (TPD) experiments were performed using an AutoChem II 2920 V5.02 chemical adsorption instrument with a thermal conductivity detector (TCD) with the heating rate of 10 ^o^C/min after the NH_3_ or H_2_ adsorption process. 0.10 g sample was loaded and pre-reduced at 200 ^o^C for 2 h in flowing Ar. After that, the temperature was cooled to 50 ^o^C, and the sample was flushed with NH_3_ or H_2_ to the saturation. Finally, the sample was ramped to target temperature, and the desorbed gas was monitored by a TCD detector. Thermogravimetric analyses were performed with TGA5500 thermal analyzer from 50 ^o^C to 800 ^o^C at a rate of 10 ^o^C min^-1^. Nitrogen sorption isotherms were obtained with Quantachrome Autosorb 1C surface area analyzer at 77 K. Pore size distribution was calculated with the Barret-Joyner-Halenda (BJH) and the surface area was obtained by Brunauer-Emmett-Teller (BET) method. X-ray photoelectron spectroscopy (XPS) was collected on a thermo ESCALAB 250 photoelectron instrument, using Al Kα radiation (1846.6 eV) at 150 W and 10.8 mA.


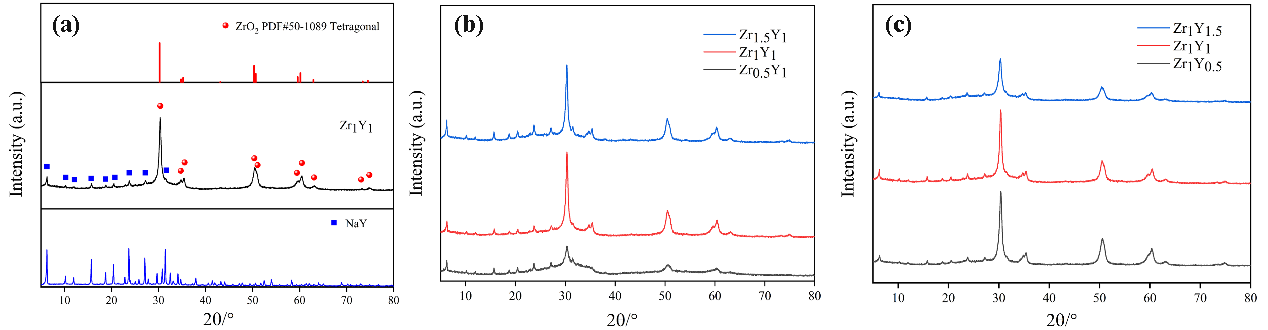


**Supplementary Figure 1.** XRD patterns of the NaY and the resulting catalysts.


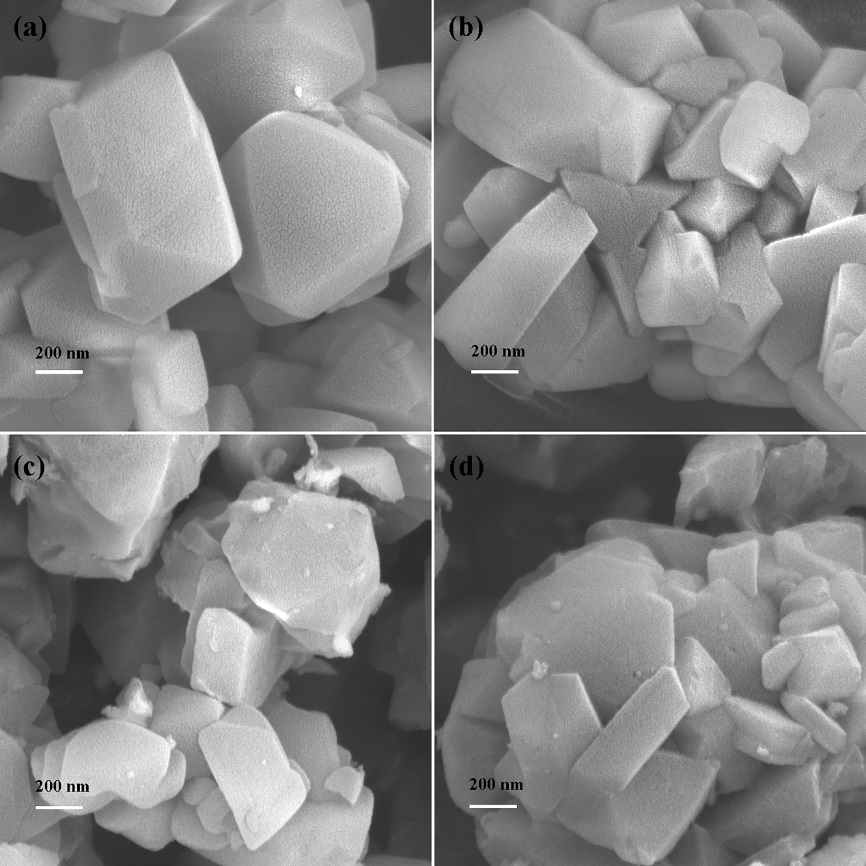


**Supplementary Figure 2.** SEM images of the NaY (a), Y_1_ (b), Zr_1_Y_1_ (c) and the used Zr_1_Y_1_ (d).





**Supplementary Figure 3.** XPS survey spectra of the Zr_1_Y_1_.


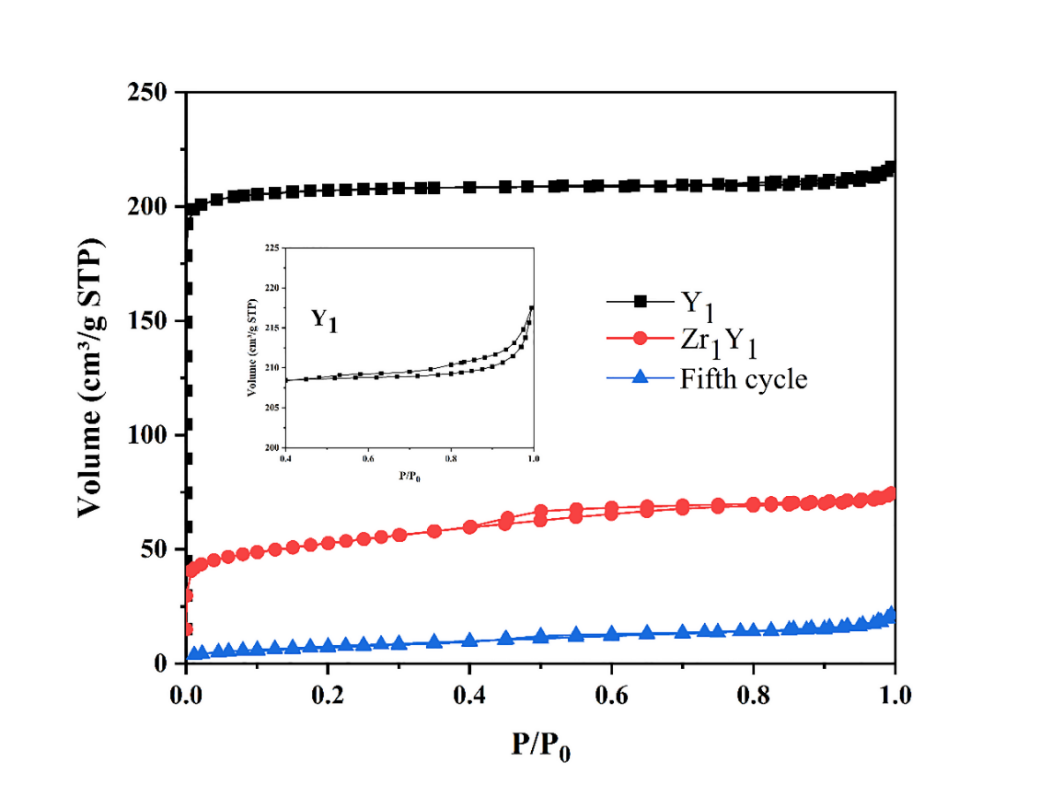


**Supplementary Figure 4.** N_2_ adsorption and desorption isotherms of the Y_1_, Zr_1_Y_1_and the used Zr_1_Y_1_.

**Supplementary Table 1.** Physical parameter of the NaY, Y_1_, Zr_1_Y_1_and the spent Zr_1_Y_1_.

|  | BET surface (m^2^/g) | Pore size (nm) | Pore volume (cm^3^/g) |
| --- | --- | --- | --- |
| NaY | 831 | 1.40 | 0.29 |
| Y_1_ | 856 | 1.54 | 0.33 |
| Zr_1_Y_1_ | 192 | 2.31 | 0.11 |
| Used Zr_1_Y_1_ | 28 | 3.76 | 0.03 |

**

**

**Supplementary Figure 5.** NH_3_-TPD results of the NaY and Zr_1_Y_1_.

**
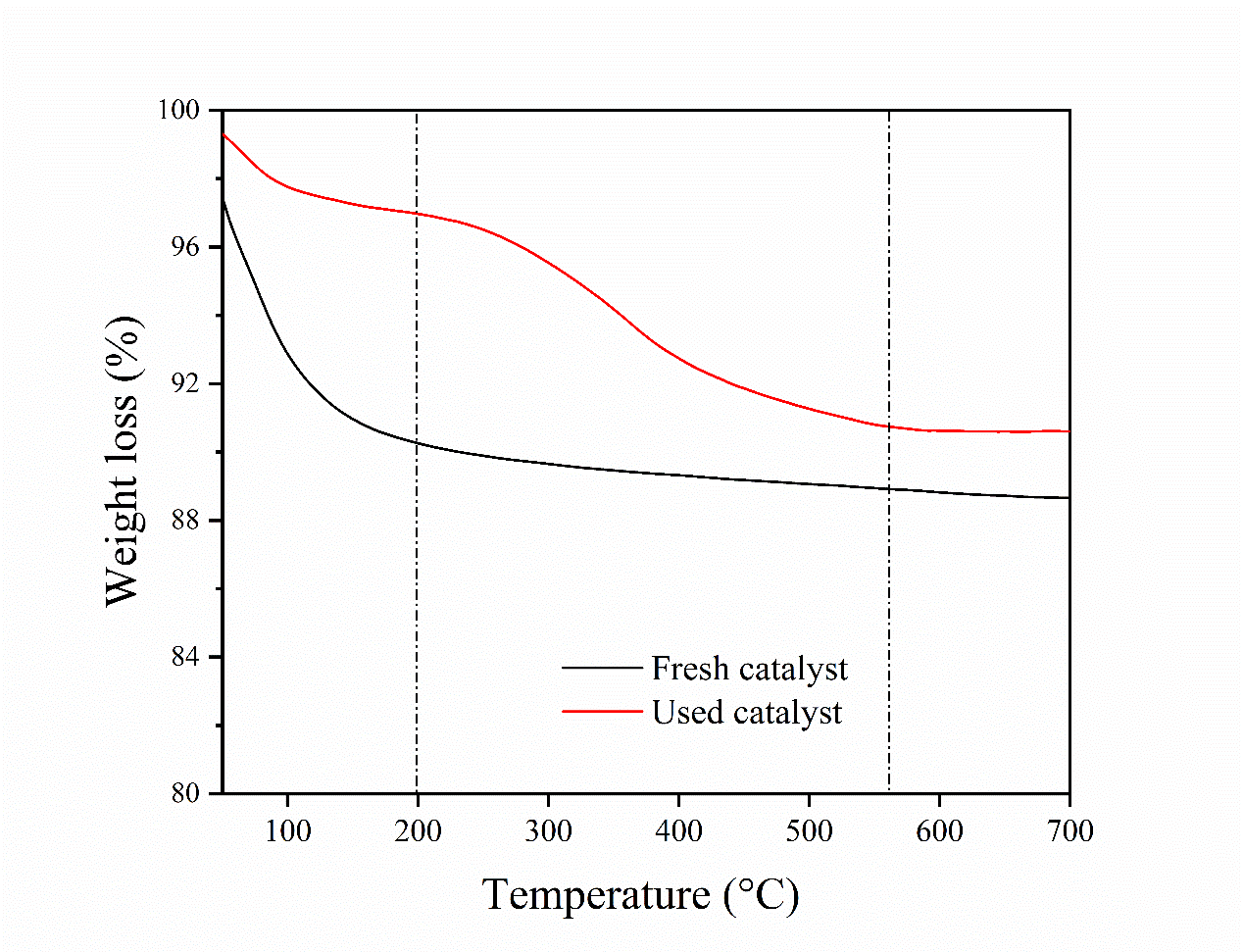
**

**Supplementary Figure 6.** TGA profiles of the Zr_1_Y_1_ and the used Zr_1_Y_1_.

**
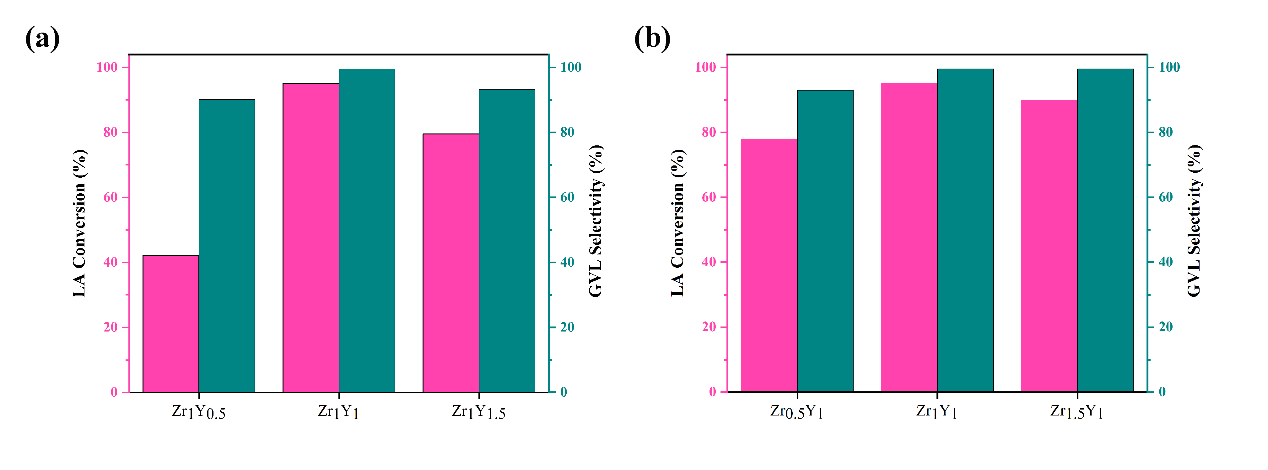
**

**Supplementary Figure 7.** Catalytic performance of the Zr_1_Y_y_ with varying dealuminzation rate and Zr_x_Y_1_ with varying Zr loading amount.


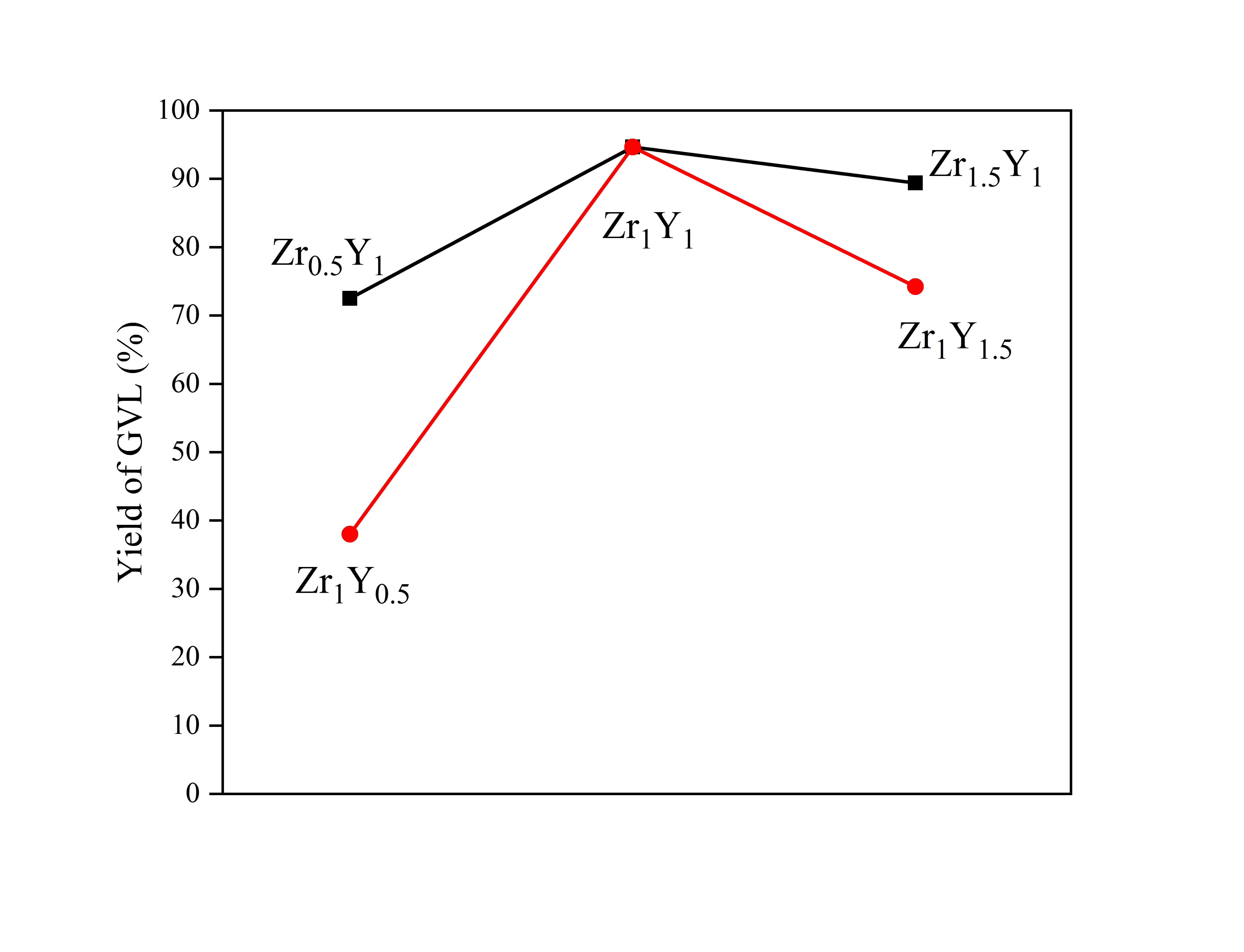


**Supplementary Figure 8.** Catalytic performance of LA hydrogenation with various catalysts.

**Supplementary Table 2.** Catalytic performance of the ZrY catalysts.

| Catalyst | Conversion of LA (%) | Selectivity to GVL (%) | Yield of GVL (%) |
| --- | --- | --- | --- |
| Zr_0.5_Y_1_ | 77.9 | 93.0 | 72.5 |
| Zr_1_Y_1_ | 95.1 | 99.5 | 94.7 |
| Zr_1.5_Y_1_ | 89.8 | 99.5 | 89.4 |
| Zr_1_Y_0.5_ | 42.2 | 90.1 | 38.0 |
| Zr_1_Y_1.5_ | 79.5 | 93.3 | 74.2 |
